# Impact of TiO_2_ Reduction and Cu Doping on Bacteria Inactivation under Artificial Solar Light Irradiation

**DOI:** 10.3390/molecules27249032

**Published:** 2022-12-18

**Authors:** Piotr Rychtowski, Oliwia Paszkiewicz, Maria Carmen Román-Martínez, Maria Ángeles Lillo-Ródenas, Agata Markowska-Szczupak, Beata Tryba

**Affiliations:** 1 Department of Catalytic and Sorbent Materials Engineering, Faculty of Chemical Technology and Engineering, West Pomeranian University of Technology in Szczecin, Pułaskiego 10, 70–322 Szczecin, Poland; 2 Department of Chemical and Process Engineering, West Pomeranian University of Technology, Piastów 42, 71–065 Szczecin, Poland; 3 Department of Inorganic Chemistry and Materials Institute (IUMA), Faculty of Sciences, University of Alicante, Carretera de San Vicente del Raspeig s/n, 03690 Alicante, Spain

**Keywords:** photocatalysis, reduced TiO_2_, Cu-TiO_2_, solar lamp irradiation, *E. coli*, *S. epidermidis*

## Abstract

Preparation of TiO_2_ using the hydrothermal treatment in NH_4_OH solution and subsequent thermal heating at 500–700 °C in Ar was performed in order to introduce some titania surface defects. The highest amount of oxygen vacancies and Ti^3+^ surface defects were observed for a sample heat-treated at 500 °C. The presence of these surface defects enhanced photocatalytic properties of titania towards the deactivation of two bacteria species, *E. coli* and *S. epidermidis,* under artificial solar lamp irradiation. Further modification of TiO_2_ was targeted towards the doping of Cu species. Cu doping was realized through the impregnation of the titania surface by Cu species supplied from various copper salts in an aqueous solution and the subsequent heating at 500 °C in Ar. The following precursors were used as a source of Cu: CuSO_4_, CuNO_3_ or Cu(CH_3_COO)_2_. Cu doping was performed for raw TiO_2_ after a hydrothermal process with and without NH_4_OH addition. The obtained results indicate that Cu species were deposited on the titania surface defects in the case of reduced TiO_2_, but on the TiO_2_ without NH_4_OH modification, Cu species were attached through the titania adsorbed hydroxyl groups. Cu doping on TiO_2_ increased the absorption of light in the visible range. Rapid inactivation of *E. coli* within 30 min was obtained for the ammonia-reduced TiO_2_ heated at 500 °C and TiO_2_ doped with Cu from CuSO_4_ solution. Photocatalytic deactivation of *S. epidermidis* was greatly enhanced through Cu doping on TiO_2_. Impregnation of TiO_2_ with CuSO_4_ was the most effective for inactivation of both *E. coli* and *S. epidermidis*.

## 1. Introduction

Among all photocatalysts, TiO_2_-based materials are the most popular due to their low cost, great stability, ease of metal doping and relatively high activity [1]. However, due to their wide band gap value, many attempts have been made to increase their light harvesting in the visible range. In addition, the bacteria inactivation mechanisms, particularly in the case of copper-modified titanium dioxide, the mechanism is still unclear and some reports indicate a synergistic antibacterial effect of Cu and photocatalytically active TiO_2_ [2]. Nevertheless, there is a need to boost TiO_2_-based materials’ activity.

Most popular modification methods among scientists are based upon the utilization of noble (Pt, Au, Ag) or transition metals (Fe, Cu), as well as the non-metal dopants such as S, F, N and C [3,4,5,6]. One of the most exceptional modification approaches is the creation of Ti^3+^ surface defects on TiO_2_, which not only increase visible light utilization, but also boost the separation of electron–hole pairs, leading to the improved formation of reactive radicals [7,8,9,10,11]. Ti^3+^ self-doping appears to be an effective way of boosting TiO_2_ antimicrobial activity in the visible range [11], whereas some reports [12] have indicated successful utilization of antimicrobial properties of Ti^3+^/Cu-modified TiO_2_ composites.

The bacteriostatic properties of copper compounds have been known for decades due to their toxicity towards pathogenic bacteria [13]. According to some reports [14], noble and semi-noble metal dopants increase the possibility of radical formation. The increase in activity is as follows: TiO_2_-Cu > TiO_2_-Au > TiO_2_-Ag > TiO_2_. However, photocatalytic inactivation of bacteria is more complex and not only focuses on reactive radical formation. For example, the photocatalytic inactivation of Gram-negative *Escherichia coli* using nanomaterials based on Cu-doped TiO_2_ proves to be an effective method, because it connects antibacterial properties of Cu and photocatalytic properties of TiO_2_, as well as facilitates charge transfer due to efficient heterojunction between both semi-conductors [2,15,16].

The most popular methods of obtaining TiO_2_-Cu composites are by using sol–gel [17,18], wet impregnation [19], magnetron sputtering [15] and thin films formation [20].

Cu-doped TiO_2_ preparation through the sol–gel method [18] showed the best results in the photocatalytic inactivation of Gram-negative bacteria *E. coli* under visible light, where 3% of Cu was introduced. It is worth mentioning that Cu doping of TiO_2_ led to improved visible light activity as well as improved stability of electron–hole pairs, followed by an increased reactive radical life-time. In the case of Gram-positive bacteria *Staphylococcus aureus*, the utilization of TiO_2_-Cu nanomaterials [21] showed long-term antibacterial properties and prevented secondary bacterial infections of orthopedics implants.

Wet-impregnated TiO_2_ with Cu salts, where 0.5 M% of copper was introduced to TiO_2_, appears to be an effective preparation method for obtaining effective photocatalysts for the disinfection of *E. coli* in river water [19]. However, the authors suggest that its high efficiency might be connected to the diffusion of Cu ions out of the titania matrix into the bacteria solution, meaning that the sample prepared in this way is most likely unstable and single-use. In order to obtain a reusable and stable photocatalyst, a preparation method in which Cu ions will be preserved in TiO_2_ matrix is required. Another important aspect is the loading of Cu-based dopants. A high percentage of copper dopants resulted in a great decrease of the specific surface area of TiO_2_, which lowers the adsorption of bacteria on reactive sites [19]. On the other hand, pH value, which directly affects PZC (point zero charge), also has a great effect on *E. coli* adsorption, leading to better photocatalytic deactivation efficiency in shorter time periods. Taking electrostatic forces into account, a lower pH value than PZC might be favored for *E. coli* adsorption. Bearing this in mind, the utilization of different Cu-based dopants will result in varied forces that will either attract bacteria into the photocatalyst porous surface or push them away.

T. Lopez and others [14] performed an in-depth XPS analysis of Cu/TiO_2_ materials. They came to the conclusion that the most promising results of *E. coli* DNA degradation are obtained when Cu^1+^ and Ti^3+^ species are present in the crystal lattice of studied composites. It is therefore important as to which oxidation state of copper the Cu/TiO_2_ composite consists of. In our previous works [8,9,10], we introduced efficient methods of Ti^3+^ formation on the surface of TiO_2_.

In this research article, we compared different TiO_2_ modification methods using reductive environment (aqueous NH_3_, gaseous NH_3_ or H_2_) versus modification with Cu-based dopants (Cu(NO_3_)_2_, CuSO_4_ or Cu(CH_3_COO)_2_), where Cu load was 1.0 wt%. All preparations were performed via high-temperature calcination. Both groups of samples were utilized in the photocatalytic deactivation of model bacteria: *Escherichia coli* (Gram-negative) and *Staphylococcus epidermidis* (Gram-positive) in the presence of artificial solar light.

## 2. Results

### 2.1. X-ray Diffraction and TEM Analyses

**Figure 1:**
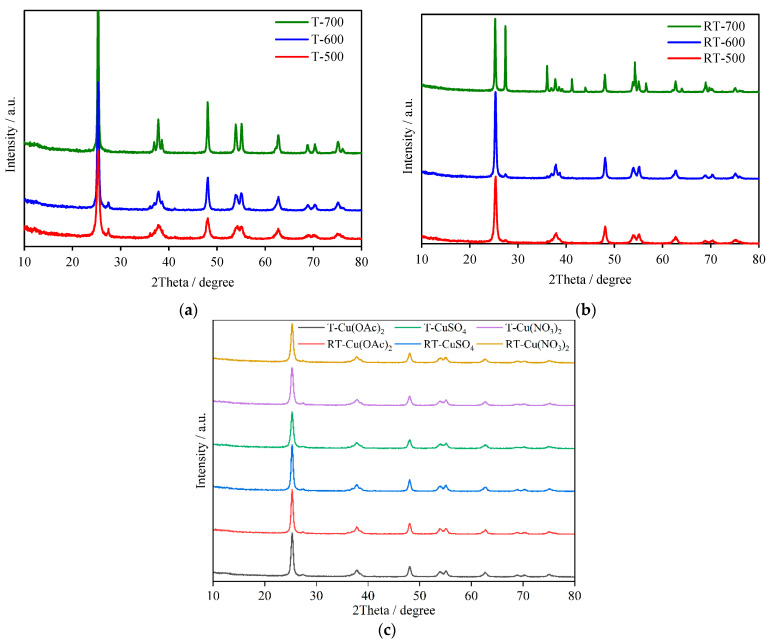
XRD diffractograms of (**a**) TiO_2_ obtained in hydrothermal process and heat-treated at 500–700 °C, (**b**) TiO_2_ obtained in hydrothermal process with NH_4_OH and heat-treated at 500−700 °C, (**c**) Cu-TiO_2_ samples.

X-ray diffractograms are presented in Figure 1. The increase in heat-treatment temperature led to an increase in the crystallinity of the anatase, as observed by the appearance of narrower and more intense reflexes. The ratio of anatase to rutile was around 96/4, respectively, and was maintained among all of the prepared samples with the exception of TiO_2_ heat-treated at 700 °C after NH_4_OH modification, which was composed from mixed phases, anatase and rutile with a ratio of 55/45, respectively. Modification of TiO_2_ with NH_4_OH accelerated its crystallization and phase transformation to rutile. In Table 1, calculated crystallite sizes for all of the studied samples based on the Scherrer equation are listed.

The structural appearance of TiO_2_ samples and Cu nanoparticles’ presence were measured via the TEM method (Figures S1–S7 in the Supplementary Materials). Both of the crystallites of anatase and rutile were visible and their size was varied from a few to over 20 nm. The nanoparticles of copper were also observed; however, their amount was low due to their low concentration in samples and very low size.

### 2.2. X-ray Fluorescence Spectroscopy

In Table 2, the resulting data from XRF measurements are presented. The raw TiO_2_ contained around 1.5 mass% of sulfur, because this material was a semi-product from the industrial production of titania white. TiO_2_ samples modified with NH_4_OH contained a lower quantity of sulfur in comparison with those which were prepared without the pretreatment of TiO_2_ with ammonia solution. Most likely, ammonia species rinsed some of the sulfate groups from the titania surface. Amounts of Cu in all the Cu-doped TiO_2_ samples were comparable.

### 2.3. Fourier-Transform Infrared Spectroscopy (FTIR)

**Figure 2:**
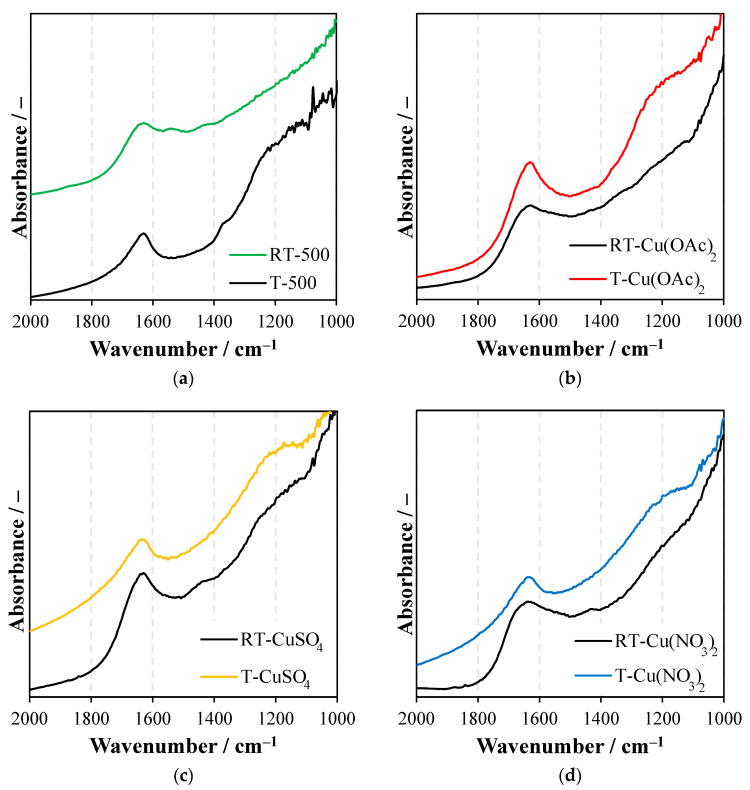
FTIR spectra of (**a**) TiO_2_ obtained in hydrothermal process with and without NH_4_OH and heat-treated at 500 °C, RT-500 and T-500, respectively; (**b**) RT-500 and T-500 treated with Cu(OAc)_2_, (**c**) RT-500 and T-500 treated with CuSO_4_, (**d**) RT-500 and T-500 treated with Cu(NO_3_)_2_.

In Figure 2a–d, FTIR spectra of prepared samples are presented.

In all the FTIR spectra there is a characteristic band at 1630–1620 cm^−1^ corresponding to the stretching vibrations of hydroxyl groups in TiO_2_ [22]. Some sulfur surface groups were identified in some of the titania samples, with a wide band at around 1240 cm^−1^ [23]. The presence of sulfur species resulted from the origin of a raw titania, which was supplied by the chemical factory (Grupa Azoty S.A. Police, Poland). A noticeable decrease in the band intensity at around 1240 cm^−1^ was observed for the titania samples reduced with NH_4_OH (Figure 2a). Most likely, some of the ammonia species such as NH_4_^+^ ions could adsorb on the sulfated titania to form ammonia sulfate, which was then removed from the surface during further treatment. However, modification of titania with CuSO_4_ (Figure 2c) caused an increase in the intensity of this band due to the naturally bounded SO_4_^2−^ species during the wet-impregnation process. Preparation of the TiO_2_ with NH_4_OH modification caused some changes in its chemical structure; two new bands were formed, at around 1440 and 1545 cm^−1^, which could be assigned to the NO_2_ stretching vibrations: symmetric and asymmetric, respectively [10]. These bands were formed upon decomposition of earlier-adsorbed ammonia groups, through the interaction of nitrogen with oxygen built in the titania lattice. A mechanism of NO_2_ formation on TiO_2_ modified by an ammonia species was described elsewhere [10]. The impregnation of TiO_2_ with Cu(OAc)_2_ (Figure 2b) caused the appearance of a new small-intensity band at the range of 1500–1400 cm^−1^, which could be assigned to some COO groups [22]. Additionally, Cu(OAc)_2_ adsorbed on the reduced titania exhibited a band at around 1620 cm^−1^ with two humps, from the left and right sides. Most likely, some of the acetate groups were adsorbed on the titania oxygen vacancy sides. Reduced titania modified with CuSO_4_ (Figure 2c) showed a new band at 1448 cm^–1^, assigned to NO vibrations, which might be formed due to the oxidation of nitrogen species on the titania surface through the sulfate anions. The same band was observed in the reduced titania modified by Cu(NO_3_)_2_ (Figure 2d). The asymmetric band at 1620 cm^−1^ in the RT-Cu(NO_3_)_2_ sample was probably a result of adsorption of Cu(NO_3_)_2_ at the titania surface defects. The adsorption of Cu(OAc)_2_ on the reduced titania led to the decrease of the O–H band at 1620 cm^−1^ and through this the surface became less hydrophilic. On the other hand, the adsorption of CuSO_4_ or Cu(NO_3_)_2_ on the reduced titania caused the opposite effect; in these cases the hydrophilicity was increased. These FTIR studies showed the impact of titania surface defects on the attachment sites of various species from the copper salts.

### 2.4. EPR Spectroscopy

**Figure 3:**
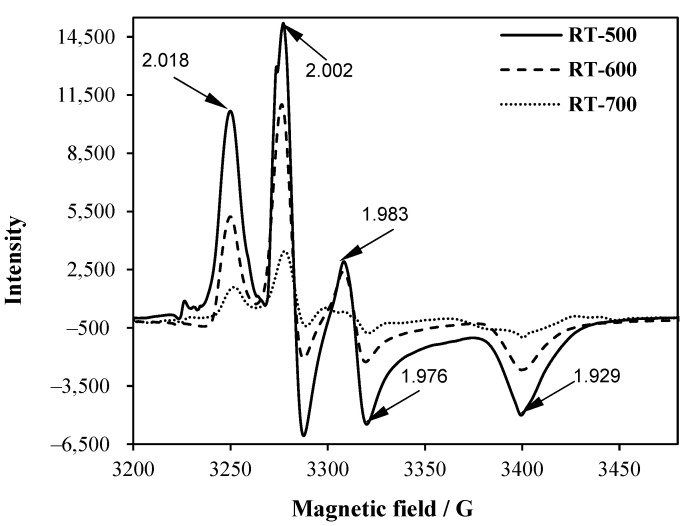
EPR spectra of reduced TiO_2_ at 500, 600 and 700 °C. Tensor g values were added.

EPR spectra of TiO_2_ samples obtained in two-step synthesis with an ammonia solution are illustrated in Figure 3. The first observed signal at 2.018 g-value refers to the surface-trapped holes (Ti^4+^O^2−^Ti^4+^O^●−^) [24]. Signals at 2.002 g-value indicated the presence of electrons trapped in oxygen vacancies, whereas a signal at 1.929 was due to the presence of Ti^3+^ in rutile [25,26]. The signal at 1.983 g-value was assigned to trapped electrons in crystal lattice [24]. All of these samples revealed similar types of defects, but their intensity varied; in general, the intensities of all the spins decreased with an increased temperature of TiO_2_ heating. So, TiO_2_ heat-treated at 500 °C exhibited the most defected structure.

### 2.5. X-ray Photoelectron Spectroscopy

XPS measurements were performed to determine the ratio of Cu_2_O to CuO species in Cu-doped TiO_2_ samples. Analyses of Ti2p and O1s binding energies were also examined and presented. The presence of inorganic residues in TiO_2_ after Cu doping from copper salts was also considered in these analyses. 

**Figure 4:**
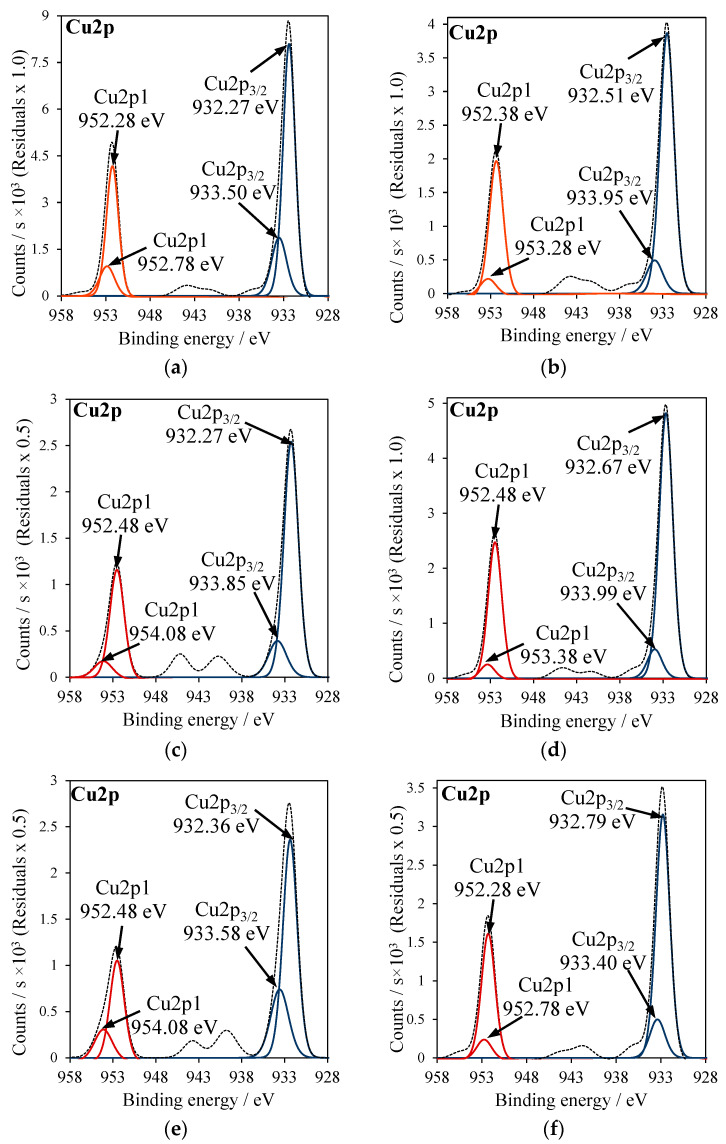
XPS spectra of Cu2p_3/2_: (**a**) RT-Cu(OAc)_2_, (**b**) RT-CuSO_4_, (**c**) RT-Cu(NO_3_)_2_, (**d**) T-Cu(OAc)_2_, (**e**) T-Cu(NO_3_)_2_, (**f**) T-CuSO_4_.

**Figure 5:**
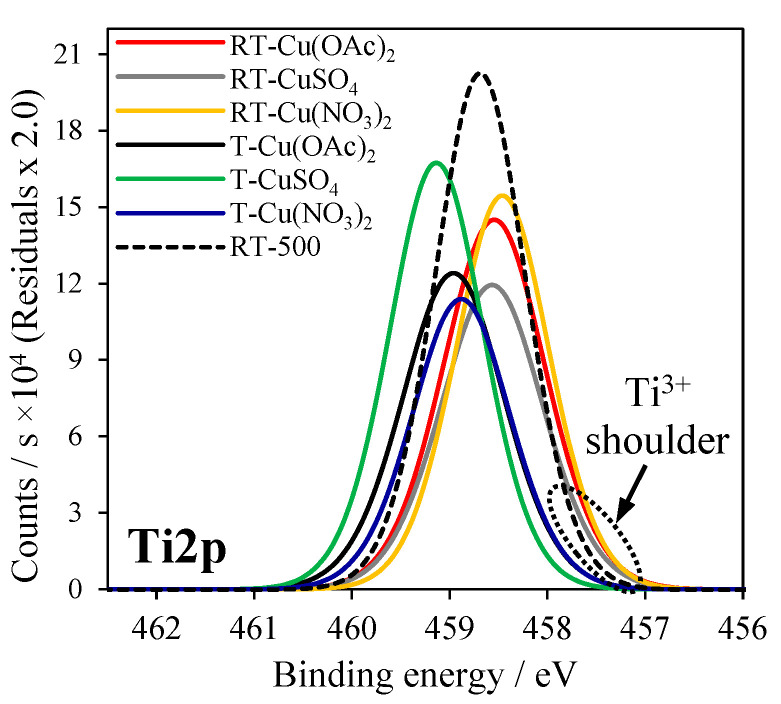
XPS spectra of Ti2p signals in RT-500 and Cu-TiO_2_ samples.

**Figure 6:**
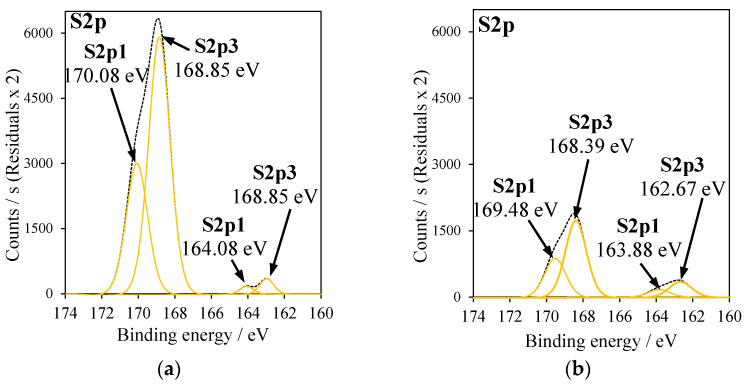
XPS spectra of S2p signals in (**a**) T-CuSO_4_, (**b**) RT-CuSO_4_.

In Figure 4, XPS spectra of Cu2p_3/2_ signals are presented, where Cu2p_3/2_ was deconvoluted into two major peaks corresponding to CuO (red line) and Cu_2_O (blue line). The calculated peak area ratios of Cu_2_O to CuO in Cu-TiO_2_ samples were listed in Table 3. In Figure 5, XPS spectra of Ti2p signals in RT-500 and Cu-doped TiO_2_ samples were added; the values of binding energies were indicated in Table 3. In Figure 6, XPS spectra for S2p3 signal in Cu-doped TiO_2_ from CuSO_4_ solution are illustrated.

The binding energy of Ti2p varied among the titania samples. The highest binding energy for Ti2p (459.14 eV) was noticed in titania modified with CuSO_4_.

According to the data reported in the literature [27], this effect can be related to the sulfur introduced to the crystal lattice of TiO_2_. However, in that studied in this research sample, it is considered that the presence of high amounts of SO_4_^2−^ bounded to the surface due to the applied method of Cu doping. Since the sulfur in this anion has a high oxidation state, the binding energy was shifted towards a higher value. A similar effect was also reported by the other researchers [28]. In the case of the RT-CuSO4 sample, the binding energy for Ti2p was lower (458.57 eV) than in the case T-CuSO_4_, because a lower quantity of sulfate groups was attached to the titania surface. In Figure 5, XPS spectra of S2p signals for T-CuSO_4_ and RT-CuSO_4_ samples are shown. Two major peaks can be observed: (1) at the binding energy of 168 eV, which refers to S^6+^ sulfur [29] and (2) at the binding energy of 163 eV, characteristic of S^2−^ [6]. It can be clearly seen that modification of TiO_2_ with an ammonia water effectively decreased the amount of sulfate species on its surface (mostly SO_4_^2−^). All the titania samples, which were reduced with NH_4_OH and doped with Cu, showed a shifting of binding energies in Ti2p signals towards lower energies (Figure 5). This could be caused by the formation of Ti^3+^ crystal lattice defects. A similar effect was reported by other researchers, who observed the presence of Ti^3+^ defects as a weak shoulder in the Ti2p peak [30,31].

**Figure 7:**
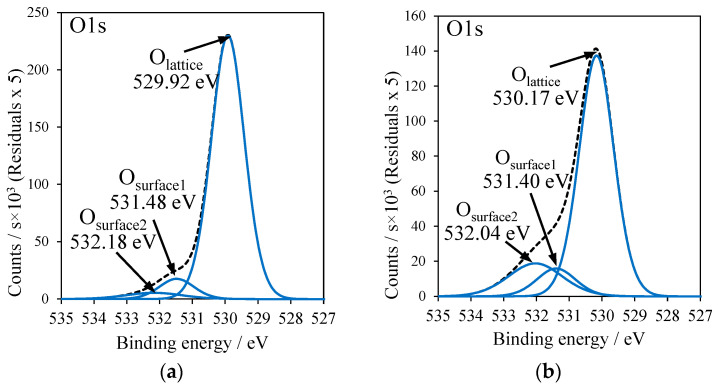

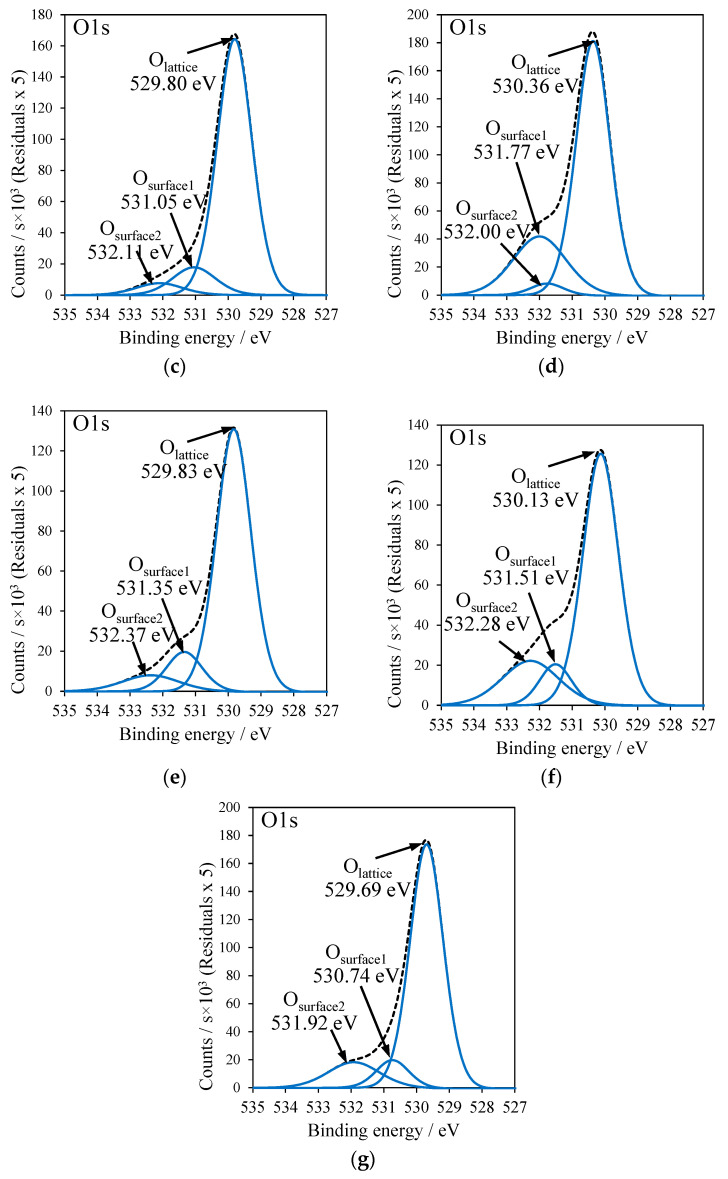
XPS spectra of O1s: (**a**) RT-500, (**b**) T-Cu(OAc)_2_, (**c**) RT-Cu(OAc)_2_, (**d**) T-CuSO_4_, (**e**) RT-CuSO_4_, (**f**) T-Cu(NO_3_)_2_, (**g**) RT-Cu(NO_3_)_2_.

In Figure 7a–g, the XPS measurements of the O1s signal are shown. This signal was asymmetric and was deconvoluted into three peaks. To facilitate the comparison of these signals, the ratio of their peak areas was calculated. The highest intensity peak, with binding energy at around 530 eV, was ascribed to the crystal lattice oxygen Ti–O of TiO_2_ (O_lattice_). The remaining signals were related to either Ti–OH species (O_surface1_) with binding energy at around 531 eV, hydroxyl groups adsorbed on the titania surface or acid residues derived from SO_4_^2−^, NO_3_^−^ or −OAc groups [32,33]. The aforementioned last signal was located with a binding energy which equaled around 532 eV (O_surface2_). First of all, the ratio of O_lattice_/O_surface_ was calculated (O_surface_ means the sum of O_surface1_ and O_surface2_) and data were introduced in Table 3. It can be clearly seen that Cu-TiO_2_ samples prepared by doping Cu to the reduced titania had a significantly higher ratio of lattice oxygen to the surface oxygen groups than the other ones. Next, O_surface1_/O_surface2_ ratios were calculated. The middle oxygen signal (O_surface1_) of Ti–OH groups most likely comes from the adsorbed hydroxyl groups present at the oxygen vacancies’ sides [32]. Therefore, Cu-TiO_2_ samples prepared by doping Cu to the reduced titania had this more intensive signal in comparison to the other ones, because the oxygen vacancies were more numerous on the reduced titania surfaces. For example, TiO_2_ reduced and treated with CuSO_4_ (RT-CuSO_4_) indicated a much higher ratio of O_surface1_/O_surface2_ (61/39) than that without preliminary reduction (T-CuSO_4_), with an oxygen surface ratio of 11/89.

### 2.6. UV-Vis Spectroscopy

**Figure 8:**
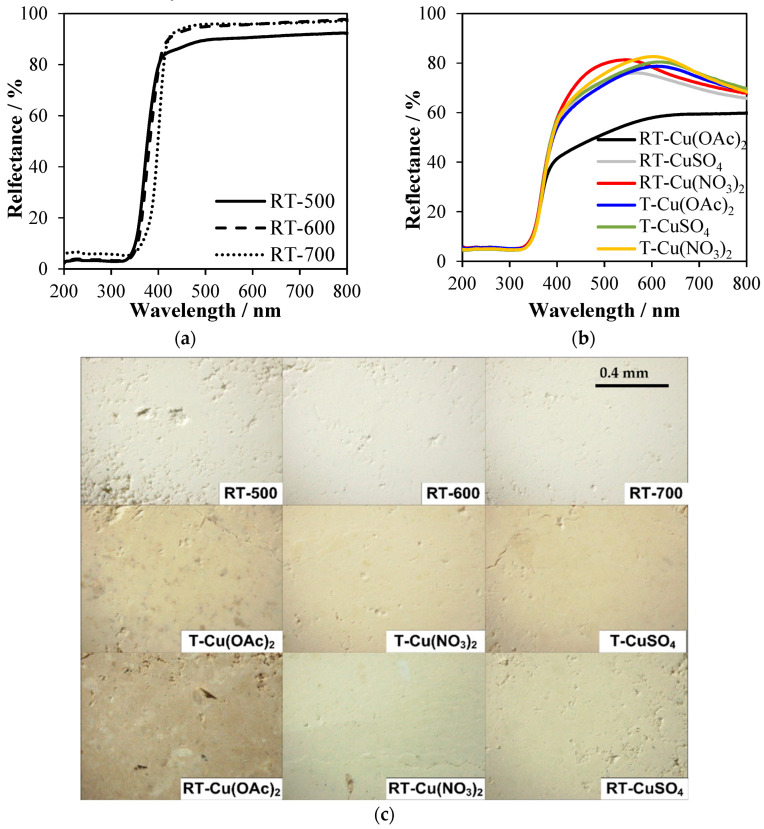
UV-Vis/DR spectra of (**a**) TiO_2_ modified with NH_4_OH; (**b**) Cu-TiO_2_; (**c**) photos of titania powders taken using an optical microscope.

UV-Vis/DR spectroscopy measurements were performed in order to determine the optical properties of studied samples. In Figure 8a, UV-Vis/DR spectra of TiO_2_ prepared upon NH_4_OH modification and following thermal heating at 500–700 °C are shown. Samples obtained at 500 °C showed enhanced absorption in the range of 390–450 nm, which could be related to the formation of some Ti^3+^ centers and oxygen vacancies. A similar effect was already observed in the other titania samples modified by NH_4_OH [34]. A TiO_2_ sample heat-treated in 700 °C (RT-700) indicated a slight shift of absorption shoulder towards the visible light range, which was caused by the formation of rutile. The modification of titania samples with Cu salts caused a significant change in the intensity of visible light absorption, as was illustrated in Figure 8b. The highest absorption in the visible range of 390–800 nm was RT-Cu(OAc)_2_, which was modified by Cu(OAc)_2_. This could be caused by the formation of carbon residues on the titania surface during carbonization of acetate groups at 500 °C. In Figure 8c, the photos of the titania powders are illustrated. The RT-Cu(OAc)_2_ sample indicated the most dark-brownish color. Reduced TiO_2_ treated with Cu(NO_3_)_2_ differs from the other titania samples in color, as it was greenish (Figure 8c). TiO_2_-Cu samples obtained from the reduced titania showed maximum reflectance at around 500 nm, whereas the Cu-TiO_2_ prepared from the titania pulp showed maximum reflectance at 600 nm. It can be concluded that TiO_2_-Cu samples, which were prepared without pretreatment with NH_4_OH, exhibited higher absorption in the visible range. This was caused by the higher quantity of oxygen surface groups adsorbed on the titania surface from the copper salts in these TiO_2_-Cu samples by comparison with those obtained from the reduced TiO_2_. These remarks are consistent with XPS analyses.

### 2.7. Zeta Potential and pH

Zeta potential was measured for titania powders suspended in an aqueous solution. Each time, the pH of aqueous suspension was measured because the acid or basic characteristics of titania surface and ionic strength could change the pH of the prepared solution. Zeta potential was measured in both saline (0.85% NaCl) and phosphate-buffered solutions to remodel the conditions used for the photocatalytic tests of bacteria inactivation. *E. coli* inactivation was carried out in a saline solution, but tests for *S. epidermidis* inactivation were performed in a phosphate buffer. Results from the measurements are presented in Table 4.

Values of zeta potential measured in 0.85% NaCl solution varied among the samples, whereas in a phosphate buffer they were very similar. A TiO_2_ sample modified with CuSO_4_ without pretreatment with NH_4_OH showed the least negative zeta potential compared with the other samples in saline solution, and the lowest pH of solution which equaled 4.4. The acidic and polar character of titania oxygen surface groups increases adsorption of water molecules and can act to enhance hydroxyl radical formation. All the samples obtained with NH_4_OH modification had a more negative potential. Cu-doped TiO_2_ samples using TiO_2_ without NH_4_OH pretreatment showed a higher acidic surface, due to the presence of a higher quantity of surface oxygen groups, as was documented using XPS analyses. However, TiO_2_ samples modified by Cu(OAc)_2_ were more hydrophobic than the other ones due to the presence of carbonized groups on the surface. These samples indicated a lower change in pH solution, but in fact their zeta potential was around (−7 mV) which was less negative than for the TiO_2_-Cu samples obtained from a Cu(NO_3_)_2_ precursor. 

### 2.8. Antimicrobial Tests towards Escherichia Coli and Staphylococcus Epidermidis Inactivation in the Presence of Solar Light

**Figure 9:**
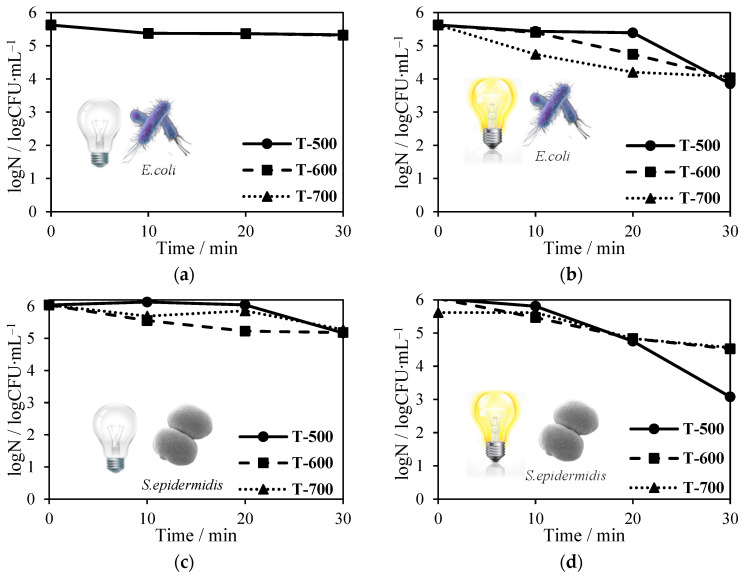
TiO_2_ antimicrobial tests on *Escherichia coli* (**a**) in the absence of solar light and (**b**) in its presence; *Staphylococcus epidermidis* (**c**) in the absence of solar light (**d**) and in its presence.

In Figure 9, the results of the microbials test for pretreatment with NH_4_OH and heat-treatment at 500–700 °C under the solar lamp and in the darkness are presented. Almost all examined photocatalysts have very poor antibacterial properties without light activation. Under artificial solar irradiation, only T-500 samples promoted bacterial reduction with counts of around 2 log CFU mL^−1^ after 30 min (Figure 9b,d). It is clear that the antibacterial activity of titania is directly correlated with its structural properties of photocatalysts. The T-500 sample is characterized by the smallest mean anatase crystallite sizes (17 nm). It is possible that such small particles penetrate inside the cells and cause an imbalance between production and accumulation of oxygen reactive species (ROS), leading to harmful effects in important cellular structures such as proteins, lipids and nucleic acids.

**Figure 10:**
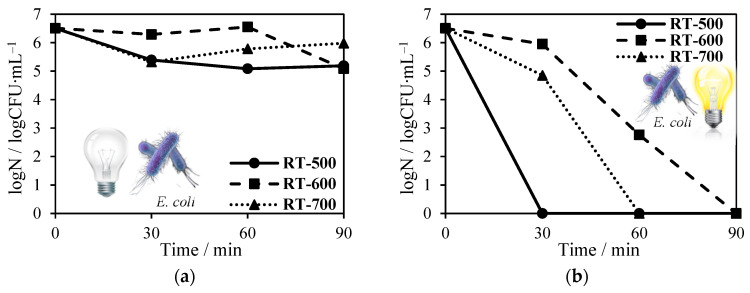

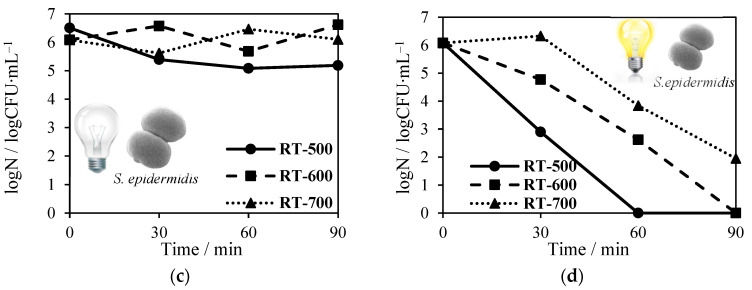
Reduced TiO_2_ antimicrobial tests on *Escherichia coli* (**a**) in the absence of solar light and (**b**) in its presence; *Staphylococcus epidermidis* (**c**) in the absence of solar light and (**d**) in its presence.

According to Marugán et al. [35], photocatalytic-process bacteria are inactivated as a consequence of the cumulative effects of serial ROS attacks on the cell membrane–wall system and internal structures, that require a sufficient time. For that reason, we decided to conduct a test for reduced TiO_2_ in a long period. The results are presented in Figure 10.

It can clearly be seen that regardless of tested photocatalyst or bacteria species, under dark conditions, even after 90 min, the decrease of bacterial number did not exceed 2 log CFU mL^−1^. The use of NH_4_OH and being heat-treated at 500, 600 and 700 °C TiO_2_ against Gram-negative *E. coli* caused total inactivation after 30, 90 and 60 min, respectively. Complete removal of Gram-positive *S. epidermidis* (6 log reduction) was attained within 90 min of the total reaction only for RT-500 and RT-600 photocatalysts.

**Figure 11:**
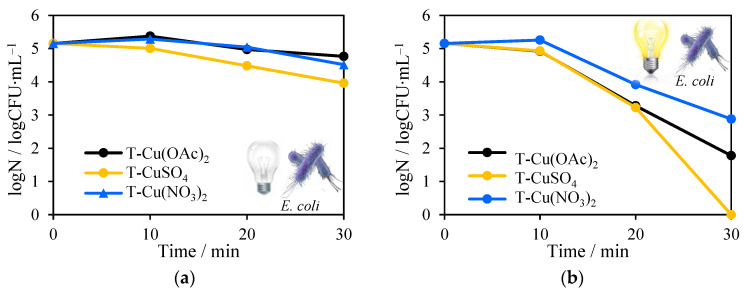

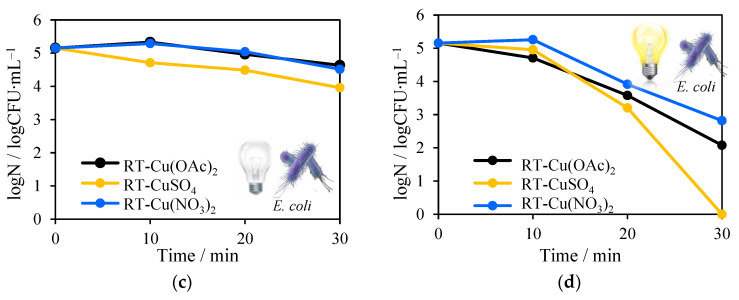
Antimicrobial tests with the addition of: Cu-TiO_2_ on *Escherichia coli* in the absence of solar light (**a**) and in its presence (**b**); reduced Cu-TiO_2_ in the absence of solar light (**c**) and in its presence (**d**).

**Figure 12:**
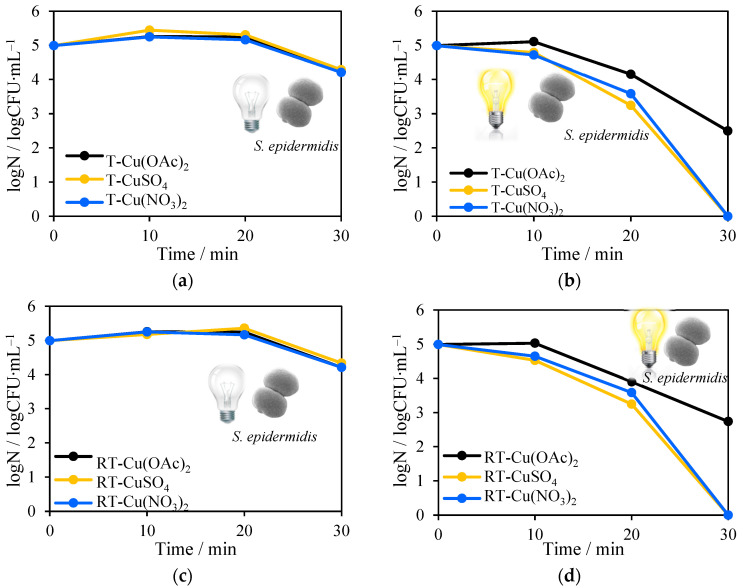
Antimicrobial tests with the addition of Cu-TiO_2_ on *S. epidermidis* in the absence of solar light (**a**) and in its presence (**b**).

**Figure 13:**
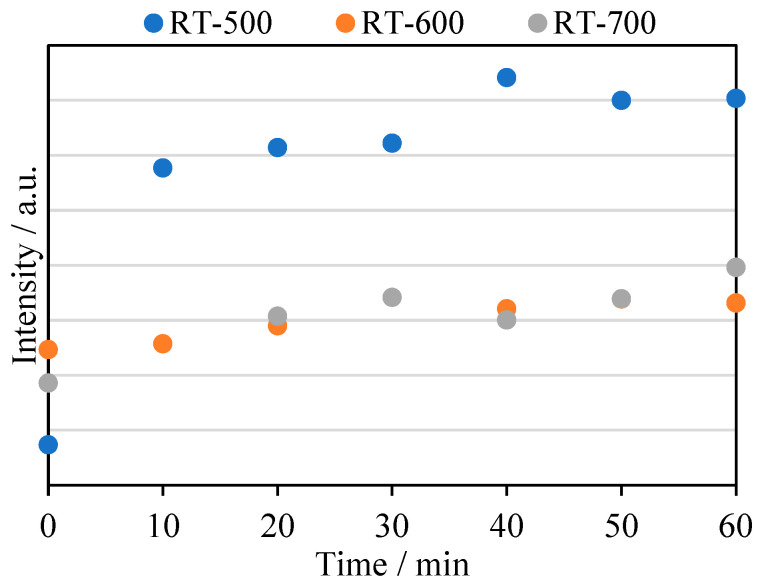
2-HTA formation in the presence of reduced TiO_2_ and ∙OH radicals.

The impregnation of NH_4_OH and being heat-treated at 500–700 °C, or reduced TiO_2_ samples with varied Cu salts, did not influence the antimicrobial properties in dark conditions (Figure 11a,c; Figure 13 a,c). The maximum 1 log reduction of the *E. coli* number was obtained for T-CuSO_4_ (Figure 11a). However, under solar irradiation testing, photocatalysts have shown the opposite results. The most promising impregnation method seems to be the use of CuSO_4_. The shortest time (30 min) was required for total inactivation of *E. coli* and *S. epidermidis* during the process, in which T-CuSO_4_ and RT-CuSO_4_ were used. The slightly lower antimicrobial efficiency was obtained for T-Cu(NO_3_)_2_ and RT-Cu(NO_3_)_2_, but only against Gram-positive *S. epidermidis* (Figure 12b,d). Unexpectedly, the worst results were from the wet impregnation with Cu(NO_3_)_2_·3H_2_O.

### 2.9. Reactive Radical Formation

In Figure 13, the detection of hydroxyl radicals formed in the presence of reduced TiO_2_ are presented. In this method, concentration of 2-hydroxyterephthalic acid was monitored as a reaction product of hydroxyl radicals and terephthalic acid during UV irradiation of TiO_2_. It can clearly be seen that the highest efficiency of hydroxyl radicals was obtained in the case of the RT-500 sample and was much higher than in the reduced TiO_2_ prepared at 600 and 700 °C. This experiment directly illustrates that the high quantity of hydroxyl radical formation on the RT-500 sample could impact on its higher antimicrobial properties compared with the other reduced TiO_2_ samples (Figure 10b,d). Moreover, the high amount of radicals formed could be strongly boosted by the presence of surface defects (see Figure 3).

## 3. Discussion

The modification of a raw titania (originally containing sulfur in its composition) with an ammonia solution in autoclave at 150 °C introduced some of the ammonia species on the titania surface. The subsequent treatment of such a prepared sample at the temperatures of 500–700 °C caused the formation of some titania surface defects in the form of oxygen vacancies and Ti^3+^ centers and electron traps at the titania lattice side. The most defected titania was obtained at 500 °C, just after the decomposition of surface-adsorbed ammonia species. At higher temperatures, rapid growing of anatase crystallites was observed and the mean size of anatase crystallites was changed from 21 to 57 nm during heat-treatment at 500 to 700 °C, respectively. The sulfate groups were also removed during the heating of titania pretreated with NH_4_OH. When we compare titania samples doped with Cu by using different copper salts with those titania reduced with ammonia and doped by using the same Cu precursors it is easy to observe the smaller quantity of sulfate species in TiO_2_ pretreated with NH_4_OH. There is a high probability that ammonia species could form the ammonia sulfate compounds on the titania surface, which were decomposed during its heating at 500 °C. The addition of copper salt to the TiO_2_ and its heating at 500 °C did not allow for the removal of sulfate species to such an extent as was noticed in TiO_2_ treated with NH_4_OH. Moreover, a higher quantity of oxygen surface groups adsorbed on the titania surface was observed in the case of Cu-doped TiO_2_ than the reduced one. However, reduced TiO_2_ doped with Cu indicated a higher quantity of OH groups adsorbed on the titania vacancy sides. The amount of Cu doped to TiO_2_ was comparable among all the samples with variation between 1.5 to 1.67 mass%. The ratio of Cu_2_O/CuO in the prepared titania samples was dependent on the copper salt used, and the highest being for CuSO_4_ and Cu(CH_3_COO)_2_. In all the TiO_2_-doped Cu samples, Cu(I) was the dominant oxidation state, and the Cu_2_O/CuO ratio varied from 72/28 to 89/11. Inactivation of two bacteria species, Gram-negative *E. coli* and Gram-positive *S. epidermidis,* was assayed with reduced TiO_2_ (RT), TiO_2_-doped Cu (T-Cu) and TiO_2_ reduced and doped with Cu (RT-Cu). Both TiO_2_ reduction and Cu doping on TiO_2_ were effective for improving the antibacterial properties of TiO_2_. However, a synergistic effect for TiO_2_ with double modification (NH_4_OH and Cu doping) was not observed. The most likely inactivation of bacteria by reduced TiO_2_ occurs through a different mechanism than in the case of TiO_2_ doped with Cu species. Copper can be very toxic for microorganisms mainly due to ‘contact killing’ mechanisms [13]. In the case of reduced TiO_2_ obtained by modification with NH_4_OH, the main route of bacteria inactivation can go through the formation of oxygen radicals on titania surfaces upon its excitation with UV light. Formed reactive radicals can oxidize outer membranes (cell wall and cell membrane) and then damage internal cellular structures. The presence of Ti^3+^ centers in TiO_2_ can increase its hydrophilicity and contribute to hydroxyl radicals’ formation, which have a very high potential for oxidation. Metallic copper can be released from the photocatalyst and can penetrate through the partially damaged membrane cells of bacteria up to cytoplasm and then cause oxidative stress and DNA degradation. These studies showed that Cu-doped TiO_2_ obtained from the CuSO_4_ precursor had the higher antibacterial potential against both species, *E. coli* and *S. epidermidis*. It is considered that some sulfate species remaining on the TiO_2_ surface could have a positive effect on increasing bacterial adhesion. According to Oh et al., many factors are responsible for this process; the most important ones include changes in the van der Waals force and electrostatic double-layer interactions or acid-base interactions, and increasing hydrophobicity [36]. 

Both the impregnation method, as well as a type of isotonic solution utilized in the experiments (NaCl or PBS), can cause changes in the nature of the obtained photocatalysts’ surface. It was shown that in 0.85% NaCl, the photocatalysts’ zeta potentials were more varied and depended on the electronic charge distribution on the photocatalyst surface, while in PBS buffer the potential differences between samples were negligible (Table 4). It is a well-known fact that the zeta potential of a surface plays an important role on the adsorption of aqueous contaminants, including bacteria, on it [37]. A higher surface charge (less negative potential) facilitates the adhesion of the bacteria to the photocatalyst, which enhances antimicrobial potential [38,39]. The electrostatic behavior of the charge-regulated surfaces of Gram-negative and Gram-positive bacteria depends on characteristic properties of cell-wall functional groups. The charge regulations allow bacteria to respond to changes in solution pH and electrolyte composition. According to Hu et al. [40], a higher *E. coli* inactivation rate was obtained at pH > 5.1. This is due to the electrostatic repulsive forces between the bacteria and the light-activated photocatalyst, which have increasing pH due to the more negative zeta potential. A similar result was obtained in our study. At a pH range of 3 to 5, more positively charged photocatalyst particles can faster diffuse to the bacteria surface and cause its inactivation. The highest activity for *E. coli* inactivation was revealed for the T-CuSO_4_ sample, with a zeta potential equal to −6.91 mV, then for T-Cu(OAc)_2_, with a zeta potential of −7.25 mV, and the worst was T-Cu(NO_3_)_2_, with a zeta potential of −8.28 mV. In the case of the *S. epidermidis* species, almost all of the titania samples showed complete inactivation of this bacteria within 30 min of solar light irradiation with the exception of the T-Cu(OAc)_2_, which contained the lowest quantity of sulfates among all the samples prepared from the titania pulp without undergoing the pretreatment process with NH_4_OH. In the case of TiO_2_-Cu-doped photocatalysts prepared from the reduced titania, bacteria inactivation was probably highly supported by the radical mechanism. It is well known that the Gram-negative *E. coli* bacteria is slightly more resistant to photocatalytic process, owing to their unique cell wall structure. This is due to the lower peptidoglycan content and lipopolysaccharide (LPS) outer membrane. It was shown that pH plays an imperative role in increasing photocatalytic efficiency under artificial solar light.

## 4. Materials and Methods

### 4.1. Materials

**Figure 14:**
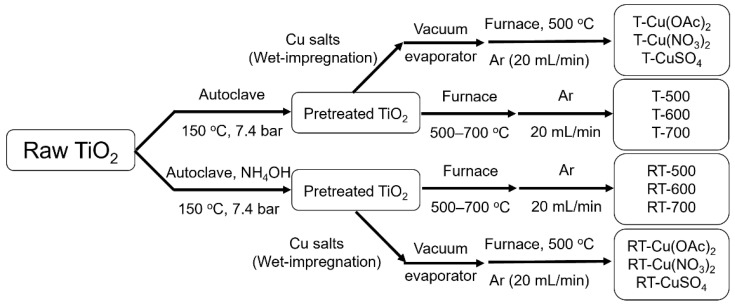
Preparation of reduced TiO_2_ (middle section) and Cu-TiO_2_ photocatalysts (outer sections).

Two-step synthesis was conducted in order to obtain reduced TiO_2_ photocatalysts. In the first step, water suspension of raw titania obtained from the chemical factory Grupa Azoty S.A. Police (Police, Poland) was heated at 150 °C under naturally increased pressure of approximately 7.4 bar for 1 h. In this step, the ammonia water solution was added to autoclave with pH regulation up to 10. In the second step, obtained pre-crystallized titania was transferred to the pipe furnace and heat-treated at either 500, 600 or 700 °C under the flow of argon (20 mL min^−1^) for 2 h. A heating rate of 10 °C/min was applied. The flowchart of TiO_2_ preparation was presented in Figure 14.

Three-step synthesis of Cu-TiO_2_ photocatalysts was conducted, where the first step was similar to that discussed previously: pre-crystallization of raw titania in a water suspension with or without the addition of ammonia water at 150 °C; 7.4 bar for 1 h. Obtained samples were then wet-impregnated with Cu(CH_3_COO)_2_·H_2_O (later marked as Cu(OAc)_2_), CuSO_4_ · 5H_2_O or Cu(NO_3_)_2_·3H_2_O water solutions in a rotary vacuum evaporator at the temperature of 60 °C and pressure of 200 mbar, until the complete evaporation of the solvent occurred. The addition of copper salt was calculated, so the final product theoretically had 1.0 wt% of Cu content. For example, to obtain a T-CuSO_4_ sample, 4 g of pre-treated TiO_2_ was mixed with 156 mg of CuSO_4_ · 5H_2_O as the sulfate group and 5 water molecules had to be taken into account. The last step of synthesis covered heat-treatment of TiO_2_ previously impregnated with Cu salt in a pipe furnace at 500 °C under the flow of argon (20 mL min^−1^) for 2 h. A heating rate of 10 °C/min was applied. The flowchart of preparation of Cu-TiO_2_ photocatalysts was presented in Figure 14.

### 4.2. Methods

Electron paramagnetic resonance (EPR) was conducted in order to define both the presence and amount of titania surface defects. EPR spectra were measured in quartz tubes under inert gas atmosphere at the temperature of −196 °C, using a JEOL JES-X310 Electron Spin Resonance Spectrometer (Tokyo, Japan).

FT-IR measurements were performed using reflection techniques in air atmosphere, using Jasco FTIR 4200 (Tokyo, Japan). Spectra were measured with a scanning speed of 1 nm/sec, resolution of 4 cm^−1^. The background was measured at first and then each time it was subtracted prior to sample measurement.

X-ray photoelectron spectroscopy (XPS) measurements were performed using Thermo-Scientific K-Alpha XPS System (Waltham, MA, USA) 1486.6 eV Al K_α_ X-ray source with a pass energy of 50 eV. A scan step of 0.1 eV was applied, irradiating 400 µm of the sample. Binding energy (B.E.) values were adjusted to the C1s transition (284.6 eV).

UV-Vis/DRS spectroscopy was used to investigate the optical properties of prepared TiO_2_ samples. These measurements were carried out in a V-650 Jasco Spectrometer (Tokyo, Japan). The spectra were recorded in the UV-Vis range of 200–800 nm (scan rate of 1 nm/s). A pure block of BaSO_4_ was used as a reference.

TEM images were taken using JEOL, JEM-2010 200 keV, with a GATAN ORIUS SC600 camera and GATAN Digital Micrograph 1.80.70 for GMS 1.8.0. Images of Cu-TiO_2_ samples have been included as supplementary materials (Figures S1–S6).

X-ray diffraction measurements (XRD) were performed with an Empyrean Diffractometer PANanalytical (Almelo, Netherlands), using a copper lamp (λ = 0.154439 nm). Measurements were carried out with the setting of Cu lamp parameters of 35 kV and 30 mA. The mean crystallite size of both anatase and rutile phases were calculated from the Scherrer Equation:

(1) $$D=\frac{K\;\cdot \;\lambda }{\left(\beta -b\right)\;\cdot \;cos\;\theta }$$

where K is the shape factor (K = 0.93), λ is the wavelength of Cu lamp (nm), β is the width of the peak at half the maximum intensity after subtraction of background (rad), b is the apparatus dilatation (rad) and θ is the diffraction angle (°).

The percentage amount of both copper and sulfur contents in TiO_2_ were measured using an energy dispersive X-ray fluorescence (EDXRF) spectrometer Epsilon3, Malvern PANanalytical, (Almelo, Netherlands), using an internal pattern.

Both zeta potential and pH measurements were performed using Malvern PANanalytical Zetasizer Nano-ZS (Almelo, Netherelands). For measurement, two types of titania suspensions were prepared. The first one was in NaCl solution with the same concentration as was used for the inactivation test of *E. coli* and the second one was in H_3_PO_4_ buffer, which was used during the inactivation test of *S. epidermidis*. Each time, 20 mg of the given photocatalyst was dispersed into 100 mL of solution, using an ultrasonic bath (15 min). The pH of suspension was measured using a pH-responsive electrode, previously calibrated by three different buffers: acidic, neutral and basic. Such prepared suspensions were transferred to the specially designed, electro-conducting measurement cells, where zeta potential was measured.

The antibacterial properties of the tested photocatalysts were determined by measuring the inactivation rate of Gram-negative Escherichia coli K12 ATCC 25992 and Gram-positive *Staphylococcus epidermidis* ATCC 49461 bacteria in the reaction suspension. The frozen bacterial cultures in 15% glycerol and appropriate freezing media were placed in a water bath at 37 °C for 5 min. Bacteria was suspended in a suitable strain liquid medium (Nutrient Broth for *E. coli* or BHI Broth for *S. epidermidis*, BioMaxima Sp. z o.o, Lublin, Poland) and incubated for 24 h at 37 °C. The solution was decanted using ultracentrifuge (5000 RPM) and the remaining bacterial pellet was diluted with a sterile isotonic saline solution containing 0.85% NaCl (Chempur, Piekary Slaskie, Poland) for *Escherichia coli* bacteria or phosphate-buffered saline PBS (Chempur, Piekary Slaskie, Poland) for Staphylococcus epidermidis. The McFarland densitometer DEN-1 (Biosan, Riga, Latvia) was used to determine the optical density OD (λ = 600 nm) of bacterial suspensions. Dilution was adjusted to 0.5 McFarland turbidity standard to achieve bacterial suspension of 1.5 × 10^8^ CFU cm^−3^. A tested photocatalyst was added to a glass bottle containing 250 cm^3^ of the started suspensions in saline or PBS buffer. The concentration of photocatalysts was 0.1 g dm^−3^. Then, 100 cm^3^ of each of the resulting suspensions of bacteria and photocatalyst were measured in glass reactors equipped with a magnetic stirrer. The entire set-up was placed on a magnetic stirrer under a UV-VIS-emitting lamp (ULTRA-VITALUX 230V E27/ES, OSRAM 300W, Munich, Germany). The stirring speed was 200 RPM. At the same time, an identically prepared experimental set was placed in an incubator without a light source (in the dark). In both cases, the temperature was maintained at 37 °C. Control experiments were carried out in the same way, without the addition of a photocatalyst, for bacterial suspensions in saline or PBS. The experiments were conducted for 30 min. In order to determine the number of viable bacteria in the reaction suspension, 0.5 cm^3^ of the reaction suspension was taken after 0, 10, 20 and 30 min. The collected sample was diluted according to the decimal dilution scheme. A saline solution (0.85% NaCl) for *E. coli* or PBS buffer for *S. epidermidis* was used to prepare the dilutions. Then, 0.25 cm^3^ of the suspensions at the appropriate concentrations were placed in Petri dishes with sterile medium Plate Count Agar (PCA, BioMaxima Sp. z o.o, Lublin, Poland) for *E. coli* or Brain Heart Infusion agar (BHI, BTL Sp. z o.o, Lodz, Poland) for *S. epidermidis*. After the application of the suspension, the plates were incubated at 37 °C for 24 h. After this time, the visible bacterial colonies were counted as log CFU mL^−1^. The results were presented as a percentage of surviving bacteria remaining after the process.

The detection of ∙OH radical formation on the TiO_2_ surface was performed using the fluorescence technique. In this method, transformation of terephthalic acid (TA) to 2-hydroxyterephthalic acid (2-HTA) was carried out in the presence of TiO_2_ and UV irradiation. The concentration of TA used was 5 × 10^−4^ mol∙dm^−3^, sample weight was 20 mg and the solution volume was 100 cm^3^. Additionally, to simulate the bacteria environment, NaCl (0.85%) was added to the reaction solution. As a source of UV light, an LED UV lamp composed of three LED diods (10 W) was used. The concentration of 2-HTA was analyzed using a fluorescence spectrophotometer (Hitachi, F-2500, Japan, Kyoto).

## 5. Conclusions

The modification of TiO_2_ with NH_4_OH and the following heat-treatment at 500–700 °C conducts the formation of some titania surface defects such as oxygen vacancies and Ti^3+^ centers, with higher quantities at high temperatures. The presence of oxygen surface defects in TiO_2_ increases its antimicrobial properties. The presence of titania surface defects increases its hydrophilicity and adsorption of hydroxyl groups, which is beneficial for OH radical formation. However, high adsorption of hydroxyl ions can change the potential charge of titania surface into negative ones. The negative zeta potential of TiO_2_ hinders its diffusion to *E. coli* species and can weaken its killing potential. The wet impregnation of TiO_2_ with copper salts and following heat-treatment at 500 °C under argon atmosphere led to the significant reduction of Cu(II) to a Cu(I) oxidation state. However, the type of copper precursor used influenced its antimicrobial properties more than the amount of Cu(I) on the TiO_2_ surface. The CuSO_4_ precursor appeared to be the best copper compound in the enhancement of antimicrobial properties of TiO_2_ in two bacteria species, *E. coli* and *S. epidermidis*. Additionally, obtained in this way, TiO_2_ photocatalyst contained the sulfur species, which could act as the toxic agent for microorganisms, as well. Moreover, such a prepared T-CuSO_4_ sample has an acidic surface and is hydrophilic with a high potential for the generation of reactive radicals. The addition of T-CuSO_4_ sample to a saline solution changes the pH into more acidic. By decreasing the pH of the solution, the zeta surface charge of TiO_2_ changes to become more positive. Therefore, it is concluded that the acidic surface, high hydrophilicity and the presence of Cu(I) in TiO_2_ increase its antimicrobial properties.

## Figures and Tables

**Table 1:** Mean crystallite size of anatase and rutile in TiO_2_ determined from the Scherrer equation (using XRD analyses).

Sample	Mean Crystallite Size/nm	
	Anatase	Rutile
Raw TiO_2_	12	19
T-500	17	70
T-600	26	51
T-700	48	–
RT-500	21	24
RT-600	30	37
RT-700	56	177
T-Cu(OAc)_2_	20	14
RT-Cu(OAc)_2_	22	23
T-CuSO_4_	16	16
RT-CuSO_4_	23	16
T-Cu(NO_3_)_2_	18	20
RT-Cu(NO_3_)_2_	19	26

**Table 2:** XRF analysis: mass content of titanium (Ti), copper (Cu) and sulfur (S).

Sample	XRF Mass Content/%		
	Ti	Cu	S
Raw TiO_2_	84.26	–	1.54
T-Cu(OAc)_2_	77.63	1.65	0.72
RT-Cu(OAc)_2_	80.70	1.62	0.11
T-CuSO_4_	77.38	1.60	1.52
RT-CuSO_4_	81.42	1.59	0.40
T-Cu(NO_3_)_2_	77.16	1.67	1.24
RT-Cu(NO_3_)_2_	80.66	1.50	0.14

**Table 3:** XPS analyses of RT-500 and Cu-TiO_2_ samples.

Sample	Cu_2_O/CuO	O_lattice_/O_surface_	O_surface1_/O_surface2_	Ti2p BE/eV
RT-500	–	89.3/10.7	66.6/33.4	458.55
T-Cu(OAc)_2_	89.3/10.7	74.5/25.5	37.7/62.3	458.96
RT-Cu(OAc)_2_	77.9/22.1	83.6/16.4	68.2/31.8	458.55
T-CuSO_4_	86.2/13.8	71.2/28.8	10.8/89.2	459.14
RT-CuSO_4_	86.7/13.3	79.7/20.3	60.8/39.2	458.57
T-Cu(NO_3_)_2_	71.9/28.1	70.1/29.9	34.4/65.6	458.88
RT-Cu(NO_3_)_2_	84.0/16.0	78.4/21.6	40.8/59.2	458.46

**Table 4:** Zeta potential and pH of Cu-TiO_2_ samples.

Sample	Solution	pH	Zeta Potential/mV
RT-500	NaCl	-	−12.43
RT-600		-	−9.15
RT-700		-	−10.78
T-Cu(OAc)_2_		5.47	−7.23
RT-Cu(OAc)_2_		6.54	−7.37
T-CuSO_4_		4.43	−6.91
RT-CuSO_4_		6.18	−8.40
T-Cu(NO_3_)_2_		4.48	−8.28
RT-Cu(NO_3_)_2_		6.47	−11.50
RT-500	H_3_PO_4_	-	−24.07
RT-600		-	−25.90
RT-700		-	−25.49
T-Cu(OAc)_2_		7.25	−24.60
RT-Cu(OAc)_2_		7.27	−24.90
T-CuSO_4_		7.24	−25.00
RT-CuSO_4_		7.24	−25.10
T-Cu(NO_3_)_2_		7.23	−24.40
RT-Cu(NO_3_)_2_		7.25	−26.00

## Data Availability

Not applicable.

## Supplementary Materials

The following supporting information can be downloaded at: https://www.mdpi.com/article/10.3390/molecules27249032/s1, Figure S1. TEM images of RT-500; Figure S2. TEM images of RT-Cu(OAc)_2_; Figure S3. TEM images of RT-CuSO_4_; Figure S4. TEM image of RT-Cu(NO_3_)_2_; Figure S5. TEM images of T-Cu(OAc)_2_; Figure S6. TEM images of T-Cu(NO_3_)_2_; Figure S7. TEM images of T-CuSO_4._

## Sample Availability

Samples of the compounds are not available from the authors.
