# Supplementary material for: Impact of TiO2 Reduction and Cu Doping on Bacteria Inactivation under Artificial Solar Light Irradiation
Source: Molecules. 2022 Dec 18;27(24):9032. doi: 10.3390/molecules27249032 (PMC9784163; doi:10.3390/molecules27249032)
Supplement: Supplementary file 1 [file molecules-27-09032-s001.zip › molecules-2081423-supplementary.pdf]

Supplementary materials

1. Selected TEM images of studied samples

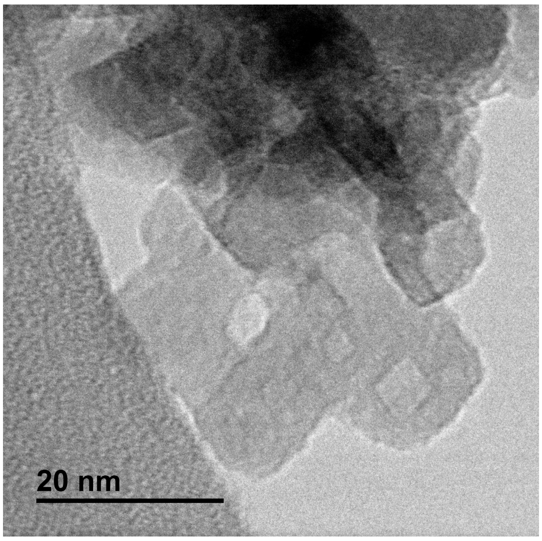

Figure S1. TEM images of RT-500

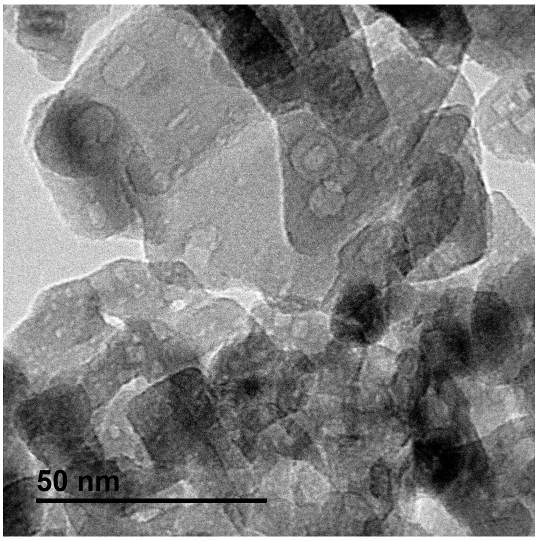

Figure S2. TEM images of RT-$Cu(OAc)_2$

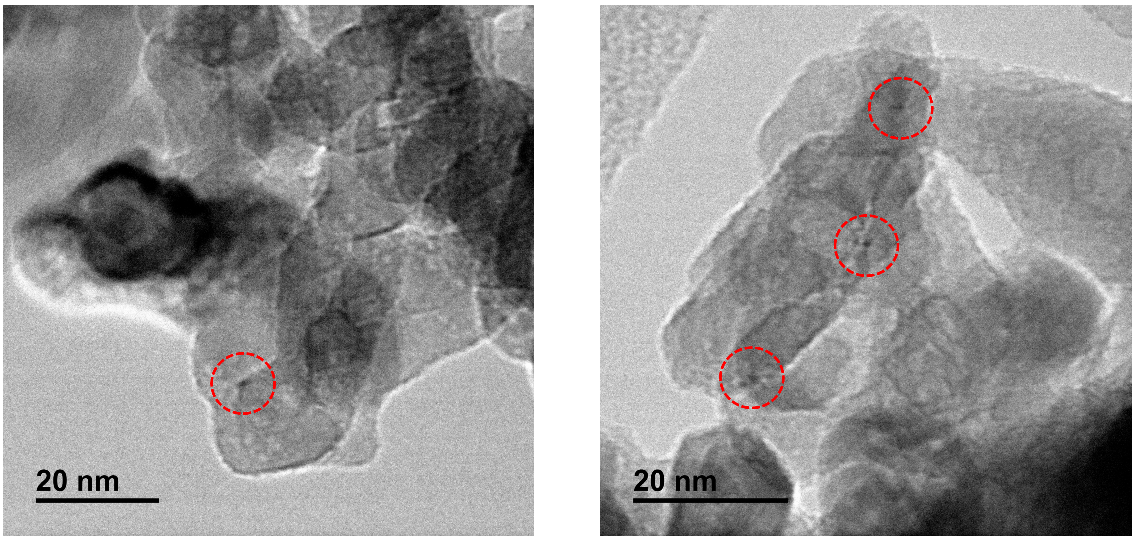

Figure S3. TEM images of RT-$CuSO_4$

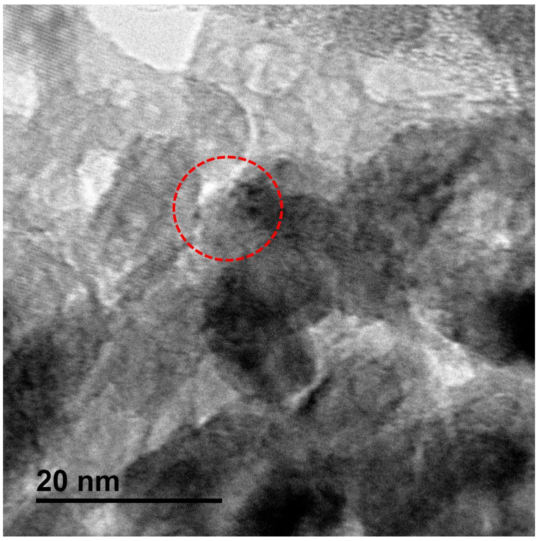

Figure S4. TEM image of RT-$Cu(NO_3)_2$

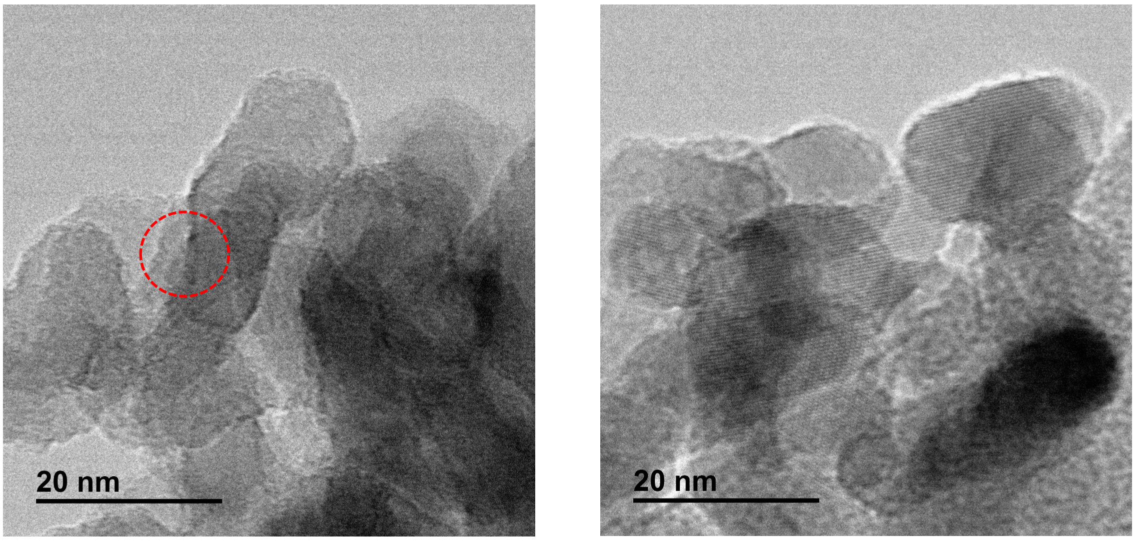

Figure S5. TEM images of T-$Cu(OAc)_2$

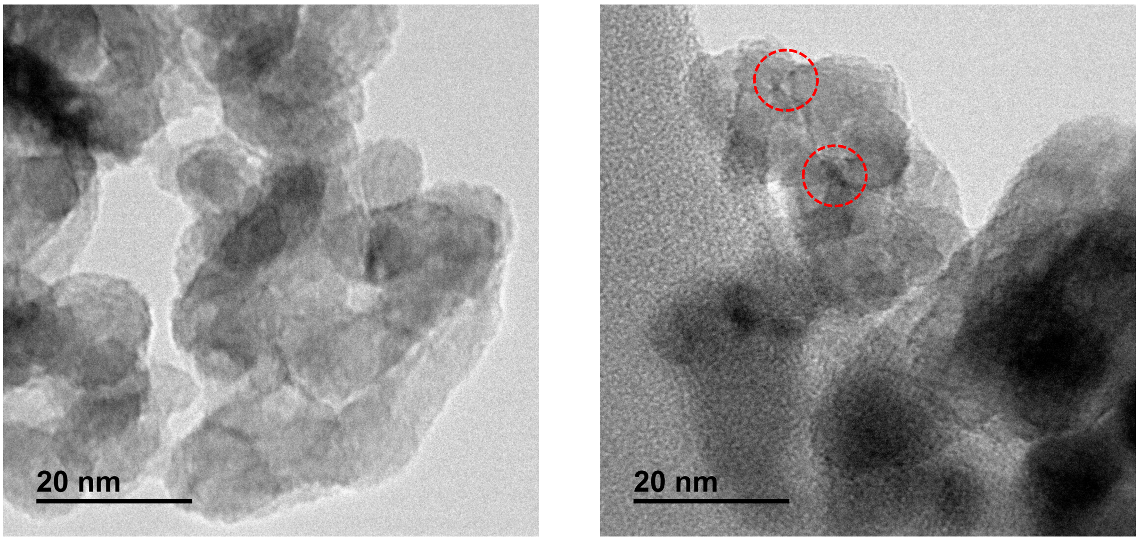

Figure S6. TEM images of T-$Cu(NO_3)_2$

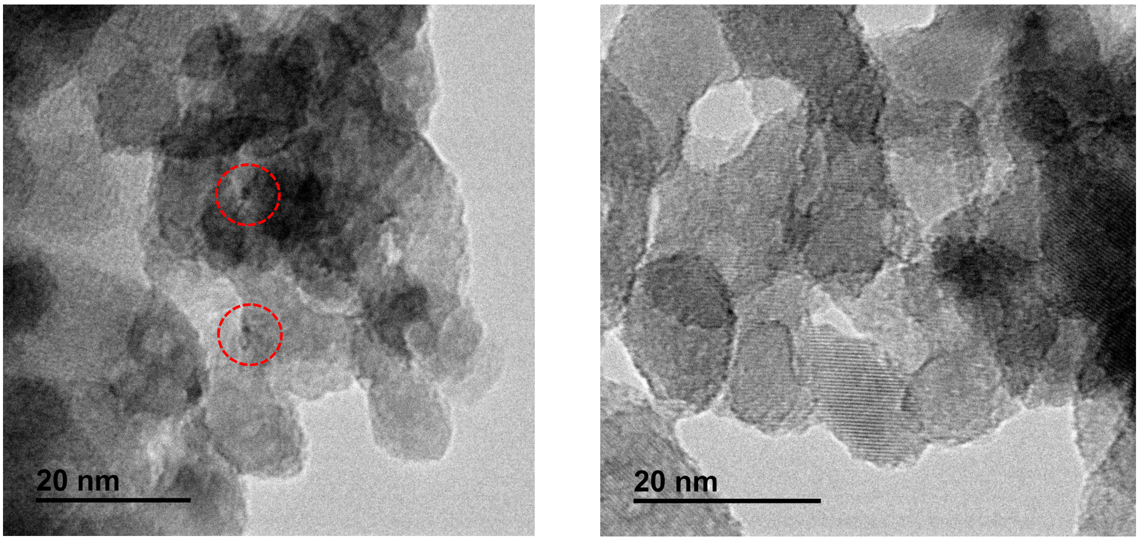

Figure S7. TEM images of T-$CuSO_4$
